# A case of doxorubicin and cyclophosphamide therapy-induced type 1 diabetes: a case report

**DOI:** 10.1186/s13256-023-03755-x

**Published:** 2023-01-27

**Authors:** Makoto Miyabayashi, Shunichiro Onishi, Tomohiko Yoshida, Minoru Takemoto

**Affiliations:** 1 International University of Health and Welfare, Narita Hospital, 852 Hatakeda, Narita City, Chiba 286-8520 Japan; 2 Department of Diabetes, Metabolism and Endocrinology, School of Medicine, International University of Health and Welfare, 4-3, Kozunomori, Narita, Chiba 286-8686 Japan

**Keywords:** Type 1 diabetes, Cyclophosphamide, Doxorubicin

## Abstract

**Background:**

Patients receiving immune checkpoint inhibitors have been reported to develop autoimmune endocrine diseases, including type 1 diabetes, although few drugs have been shown to induce type 1 diabetes. Additionally, it is important to note that drugs other than immune checkpoint inhibitors could lead to the development of type 1 diabetes.

**Case presentation:**

A 54-year-old Filipino female patient underwent surgery for left-sided breast cancer. Postoperative chemotherapy was initiated, including doxorubicin (Adriamycin) and cyclophosphamide therapy. The patient was brought to our hospital by ambulance after consciousness disturbance following three courses of doxorubicin and cyclophosphamide therapy and was hospitalized. Her blood glucose and hemoglobin A1c levels were 1661 mg/dL and 11.9%, respectively. The patient was diagnosed with diabetic ketoacidosis after arterial blood gas analysis indicated a blood pH of 7.120. Her insulin secretion was impaired, and her anti-glutamic acid decarboxylase antibody test result was significantly positive.

**Conclusions:**

The present case shows that doxorubicin and cyclophosphamide therapy may cause unexpected adverse responses, such as type 1 diabetes, though rarely, and highlights the importance of careful patient follow-up. This report is the first to present a case of type 1 diabetes that suddenly developed after doxorubicin and cyclophosphamide treatment.

## Background

In Japan, type 1 diabetes is relatively rare compared with type 2 diabetes [1]. However, the etiology of type 1 diabetes remains insufficiently understood. Viral infection such as enterovirus infection reportedly initiates beta-cell dysfunction [2, 3]. Furthermore, nude mice do not develop diabetes [4]; thus, T-cell immune system activation might be crucial for the development of type 1 diabetes [5]. Other factors such as increased intestinal permeability [6], higher-gluten diet [7], decreased beta-cell sulfatide content [8], and genetic disposition might also be involved in the development of type 1 diabetes.

Patients receiving immune checkpoint inhibitors have been reported to develop autoimmune endocrine diseases, including type 1 diabetes, although few drugs have been shown to induce type 1 diabetes [9]. Additionally, it is important to note that drugs other than immune checkpoint inhibitors could lead to the development of type 1 diabetes.

Here, we report a case of type 1 diabetes triggered by doxorubicin (Adriamycin) and cyclophosphamide therapy (AC therapy) in a patient with breast cancer.

This case has taught us the importance of checking blood glucose when using AC therapy to diagnose type 1 diabetes promptly.

## Case presentation

A patient (54-year-old Filipino woman) underwent surgery for left-sided breast cancer in February 2021. The patient had no history of autoimmune diseases. She took no regular medicine and was married to a Japanese man. She had one daughter (one pregnancy and one gravidity). Currently, she is a middle-class housewife. She stopped smoking 2 years ago (five cigarettes per day) and does not consume alcohol. Postoperative chemotherapy was then initiated, including peripheral 91.2 mg (60 mg/m^2^) of doxorubicin (Adriamycin) and 912 mg (600 mg/m^2^) of cyclophosphamide (AC) therapy intravenously. The patient claimed to have not experienced diabetes in the past but developed anorexia after the third cycle of AC therapy, which was administered on 7 May. She manifested polydipsia, polyuria, and weight loss. She had no recent infection or vaccination. On 11 May, the patient was brought to our hospital by ambulance after disturbance of consciousness and was hospitalized. Findings on admission included a body height of 148 cm, weight of 55.2 kg, body mass index of 25.2 kg/m^2^, Glasgow Coma Scale score of 8 (E2V2M4), body temperature of 36.3 °C, blood pressure of 80/56 mmHg, and a pulse rate of 108 beats per minute.

Physical examination revealed the following: the bulbar conjunctiva was slightly pale but not icteric; there was no goiter, the skin was dry, and the skin turgor was reduced. There was no abnormality on lung and heart auscultation; the abdomen was soft and flat, and there was no lower leg edema. Neurological examination revealed that the tendon reflexes were normal, and no pathological reflex was observed. Light and radial reflection was normal. Mental status, motor function, and sensory assessments could not be performed due to her low consciousness on admission.

Laboratory findings included increased urinary glucose and urinary ketone as well as increased blood ketone body levels. Her casual blood glucose and hemoglobin A1c (HbA1c) levels were 1661 mg/dL and 11.9%, respectively. Arterial blood gas analysis revealed a blood pH of 7.120, and the patient was diagnosed with diabetic ketoacidosis. Her insulin secretion was impaired and anti-glutamic acid decarboxylase (anti-GAD) antibody test result was significantly positive (> 2000 IU/ml).

After the onset of type 1 diabetes, we examined the anti-thyroglobulin and antiperoxidase antibodies, considering that this type of diabetes is sometimes complicated with an autoimmune thyroid disease. Nonetheless, these antibodies were negative in our patient. Upon admission (day 1), 60 mL per hour of Ringer’s lactate solution and 60 mL per hour of a solution containing 35 mEq/L of Na, 20 mEq/L of K, 35 mEq/L of Cl, and 20 mEq/L of lactate, and one ampule of multivitamin, were administered intravenously. A continuous insulin infusion (3.5 U per hour) was started, and the amount of insulin delivered was adjusted according to the blood glucose levels every 2 hours during her intensive care unit (ICU) stay. To avoid stress-induced gastric ulcers and secondary infections, intravenous famotidine (20 mg) was administered once daily, and 2.25 mg of tazobactam piperacillin hydrate was administered every 8 hours. Her consciousness was gradually restored. She was moved to the general hospital beds from the ICU 4 days after the onset of diabetes ketoacidosis. The continuous insulin infusion and drip infusion therapy were stopped on day 4, and insulin aspart was administered at a dose of 12 units before breakfast, 4 units before lunch, and 6 units before dinner. Additionally, 18 units of insulin degludec were used before going to bed. The patient was finally discharged 18 days after hospitalization.

After hospitalization, she continued to undergo treatment with intensive insulin therapy. Six months after hospitalization, insulin aspart was administered at a dose of 6 units before breakfast, 6 units before lunch, and 4 units before dinner. Additionally, 16 units of insulin degludec were used before going to bed. Her HbA1c and body weight improved to 6.6% and 57 kg, respectively (Table 1).

**Table 1:** Laboratory findings on admission as well as 6 months after hospitalization

	On admission	6 months after hospitalization	Normal range
WBC (/μ)	14,300	4980	3300–8600
RBC (×10^6^/μ)	4.32	4.72	3.86–4.92
Hb (g/dL)	12.5	12.7	11.6–14.8
Hct (%)	36.2	39.3	35.1–44,4
Platelets (×10^3^/μ)	121.0	231	158–348
Blood glucose (mg/dL)	1161	86	73–109
HbA1c (%)	11.9	6.6	4.9–6.0
AST (U/L)	30	18	13–30
ALT (U/L)	23	18	7–23
γ-GT (U/L)	61	23	9–32
LDH (U/L)	461	154	124–222
T-bilirubin (mg/dL)	0.4	0.3	0.4–1.5
ALP (U/L)	188	60	38–113
BUN (mg/dL)	88.1	14.7	8–20
Cre (mg/dL)	2.14	0.88	0.46–0.79
Na (mEq/L)	152	143	138–145
K (mEq/L)	4.3	4.7	3.6–4.8
Cl (mEq/L)	110	105	101–108
Acetoacetic acid (μmol/L)	562	NA	− 55
3-Hydroxybutyric acid (μmol/L)	1208	NA	− 85
Total ketone bodies (μmol/L)	1770	NA	− 130
CRP (mg/dL)	9.82	NA	0.0–0.2
U-ketone bodies	(1+)	(−)	(−)

*HLA* human leukocyte antigen

## Discussion and conclusions

Herein, we reported our experience with a 54-year-old woman who developed acute onset of type 1 diabetes, possibly triggered by doxorubicin and cyclophosphamide (AC) therapy, which was administered as postoperative chemotherapy for breast cancer. To the best of our knowledge, this is the first report describing AC therapy-induced type 1 diabetes; thus, this case might be unique.

In the field of anticancer therapy, it is known that immune checkpoint inhibitors often cause type 1 diabetes, known as immune-related adverse events. Therefore, most clinicians have paid attention to not only blood glucose but also endocrine hormones when they use immune checkpoint inhibitors. Doxorubicin and cyclophosphamide have also been widely used but have seldom been reported to lead to the development of type 1 diabetes. Our case might indicate that doxorubicin and cyclophosphamide cause type 1 diabetes. In the present case, the HbA1c level was 11.9% on admission, while it was 5.9% before breast cancer surgery. Nevertheless, neither fasting blood glucose (FBS) nor HbA1c was measured after the first or the second AC therapy. Therefore, blood glucose should be monitored when administering doxorubicin and/or cyclophosphamide.

Acute-onset type 1 diabetes is considered to develop due to multiple factors, including genetic factors such as the human leukocyte antigen (HLA) and environmental factors. In the present case, as demonstrated in Table 2, the patient originated from the Philippines where DR3 and DR9 genes are associated with disease susceptibility [10].

**Table 2:** The HLA type in the present patient

HLA-A	02:01:01	11:01:01
HLA-B	15:35	48:01:01
HLA-C	07:02:01	08:01:01
HLA-DRB1	09:01:02	15:02:01
HLA-DRB3/4/5	4 × 01:03:02	5 × 01:01:01
HLA-DQA1	01:02:01	03:02:01
HLA-DQB1	03:03:02	05:02:01
HLA-DPA1	02:02:02	–
HLA-DPB1	05:01:01	135:01

*WBC* white blood cell, *RBC* red blood cell, *Hb* hemoglobin, *Ht* hematocrit, *HbA1c* hemoglobin A1c, *AST* aspartate aminotransferase, *ALT* alanine aminotransferase, *g-GT* gamma-glutamyl transferase, *T-bilirubin* total-bilirubin, *ALP* alkaline phosphatase, *BUN* blood urea nitrogen, *Cre* creatinine, *CRP* c-reactive protein, *U-ketone bodies* urinary ketone bodies

Cyclophosphamide reportedly induces autoimmune diabetes in non-obese diabetic (NOD) mice as it decreases suppressor T cells, such as regulatory T cells (Tregs), while enhancement of proinflammatory T-helper type 1 response induced by cyclophosphamide presumably underlies the development of autoimmune diabetes [11]. In humans, only one case has been reported previously. HLA types in the previously reported case (DRB1 × 03DQB1 × 02/DRB1 × 13DQB1 × 0604) were different from the ones in the present case [12]. On the basis of the results from studies in NOD mice, insulitis precedes the onset of autoimmune diabetes. Nevertheless, in the present study, this type of insulitis could not be detected before the development of type 1 diabetes. Cyclophosphamide therapy has been reported to improve kidney function in patients with IgA nephropathy but increased the likelihood of new onset of diabetes [13]. The new onset of diabetes was probably caused by the increased body weight. Therefore, cyclophosphamide might increase the incidence of not only type 2 diabetes but also type 1 diabetes, as in our case. Is has been reported that doxorubicin causes myocardial injury known as doxorubicin-induced cardiomyopathy [14]. Although doxorubicin-induced type 1 diabetes has not been previously described, the possibility that doxorubicin might have a role in the development of type 1 diabetes in the present study could not be excluded. Therefore, this report might be the second case of cyclophosphamide-induced type 1 diabetes or the first case of doxorubicin-induced type 1 diabetes.

Given that her HbA1c before starting AC therapy was 5.9%, she might have prediabetes with an autoimmune process, such as latent autoimmune diabetes in adults. Thus, checking the autoantibodies that are known to cause type 1 diabetes is recommended before starting AC therapy.

Many reports have documented type 1 diabetes in patients receiving immune checkpoint inhibitors [9]. The results of the present case indicate that AC therapy may cause unexpected adverse reactions, though rarely, and requires thorough patient follow-up. Investigation of additional patients is required to reach more concrete conclusions.

## Data Availability

Data are available upon request.
